# Metabolic syndrome in Thai schizophrenic patients: a naturalistic one-year follow-up study

**DOI:** 10.1186/1471-244X-7-14

**Published:** 2007-04-23

**Authors:** Manit Srisurapanont, Surinporn Likhitsathian, Vudhichai Boonyanaruthee, Chawanun Charnsilp, Ngamwong Jarusuraisin

**Affiliations:** 1 Department of Psychiatry, Faculty of Medicine, Chiang Mai University, Amphur Muang, Chiang Mai, 50200 THAILAND

## Abstract

**Background:**

Not only the prevalence, but also the progress of metabolic abnormalities in schizophrenic patients is of importance for treatment planning and policy making. However, there have been very few prospective studies of metabolic disturbance in schizophrenic patients. This study aimed to assess the progress of metabolic abnormalities in Thai individuals with schizophrenia by estimating their one-year incidence rate of metabolic syndrome (MetS).

**Methods:**

We screened all schizophrenic patients who visited our psychiatric clinic. After the exclusion of participants with MetS at baseline, each subject was reassessed at 6 and 12 months to determine the occurrence of MetS. The definition of MetS, as proposed by the International Diabetes Federation (IDF), was applied.

**Results:**

Fifty-seven participants (24 males and 33 females) had a mean of age and duration of antipsychotic treatment of 37.5 years old and 8.4 years, respectively. At baseline, 13 subjects met the MetS definition. Of 44 subjects who had no MetS at baseline, 35 could be followed up. Seven of these 35 subjects (20.0%) had developed MetS at the 6- or 12-month visit, after already having 2 MetS components at baseline. The demographic data and characteristics of those developing and not developing MetS were not different in any respect.

**Conclusion:**

Thai schizophrenic patients are likely to develop MetS. Their metabolic abnormalities may progress rapidly and fulfill the MetS definition within a year of follow-up. These findings support the importance of assessing and monitoring metabolic syndrome in schizophrenic patients.

## Background

During the last several years, there has been a growing interest in metabolic abnormalities in the general population and schizophrenic patients. These disturbances are highly coincident and called metabolic syndrome (MetS), which is a cluster of risk factors for cardiovascular disease comprising central obesity, dyslipidemia, hypertension, and elevated fasting plasma glucose (FPG) level. The results of a recent meta-analysis of 21 studies showed that individuals with the MetS, compared to those without, had an increased mortality from all causes (relative risk [RR] 1.35; 95% confidence interval [CI], 1.17–1.56) and cardiovascular disease (RR 1.74; 95% CI, 1.29–2.35); as well as an increased incidence of cardiovascular disease (RR 1.53; 95% CI, 1.26–1.87), coronary heart disease (RR 1.52; 95% CI, 1.37–1.69) and stroke (RR 1.76; 95% CI, 1.37–2.25) [1].

There has been a number of MetS definitions presented over the last decade. The widely accepted ones are those proposed by i) National Cholesterol Education Program Adult Treatment Panel III (ATP III) [2]; ii) American Heart Association/National Heart, Lung, and Blood Institute, AHA/NHLBI (updated ATP III) [3]; and iii) International Diabetes Federation (IDF) [4]. Table 1 shows the similarities and differences among these definitions. A major difference between the ATP and IDF diagnostic systems is the necessity of central obesity for making a diagnosis. While the updated ATP III definition requires any three of five criteria for a diagnosis, the IDF definition needs central obesity plus any other two abnormalities. Despite this difference, updated ATP III and IDF criteria still identify essentially the same individuals as having MetS. In addition, recommendations for clinical management are virtually identical in the updated ATP III and IDF reports [3].

**Table 1:** ATPIII, Updated ATP III, and IDF definitions of metabolic syndrome [2-4]

	**ATP III (2000)**	**Updated ATP III (2004)**	**IDF (2005)**
Elevated waist circumference	Male ≥ 102 cm; Female ≥ 88 cm	Male ≥ 102 cm; Female ≥ 88 cm for Caucasians	
		
		Male ≥ 90 cm; Female ≥ 80 cm for Asian Americans	Male ≥ 90 cm; Female ≥ 80 cm for South Asians/Chinese

Elevated triglycerides	≥150 mg/dL	≥150 mg/dL or receiving drug treatment	

Decreased high-density lipoprotein cholesterol	Male <40 mg/dL; Female <50 mg/dL	Male <40 mg/dL; Female <50 mg/dL or receiving drug treatment	

Elevated blood pressure	≥130/≥85 mm Hg	≥130/≥85 mm Hg or receiving drug treatment	

Elevated fasting plasma glucose	≥110 mg/dL	≥100 mg/dL or receiving drug treatment	

Depending on the various definitions used, prevalence studies of MetS in large sample sizes of schizophrenic patients showed rates of between 28.4% and 44.7%. In 240 Canadian subjects with schizophrenia or schizoaffective disorder, 44.7% of them met the ATP III diagnosis of MetS [5]. Both the original and updated ATP III definitions were applied in 689 Americans with schizophrenia, who participated in the Clinical Antipsychotic Trials of Intervention Effectiveness (CATIE) Schizophrenia Trial. The prevalence rates of MetS in the CATIE study were 40.9% (ATP III) and 42.7% (updated ATP III) [6]. A more recent study in 430 Belgian subjects with schizophrenia showed a prevalence of 28.4% (ATP III), 32.3% (updated ATP III), and 36% (IDF) [7].

While the prevalence of MetS in schizophrenic patients is approximately two to four times higher than that in the general population [6,8], people with MetS are twice as likely to die from it and three times more likely to have a heart attack or stroke compared to people without it [9], These findings are in concordance with the fact that cardiovascular disease ranks high among the natural causes of death in the schizophrenia population [10,11].

There has been very little evidence about the prevalence of MetS in Thai people. The limited data have shown that 18.5%–26.5% of Thai people may have ATP III MetS in comparison to 30.9%–35.3% of the US population [2]. To our knowledge, there has been no prevalence study of MetS in any Asian psychotic population.

Not only the prevalence, but also the progress of metabolic abnormalities in schizophrenic patients is of importance for treatment planning and policy making. However, there have been very few prospective studies of metabolic disturbance in schizophrenic patients. We, therefore, proposed an assessment of metabolic abnormality progress in Thai individuals with schizophrenia by conducting a one-year prospective study of MetS in this population.

## Methods

### Study location

We carried out this study at a psychiatric clinic in Chiang Mai University Hospital, Chiang Mai, Thailand; a general hospital providing tertiary care for people in northern Thailand. The study was approved by the Ethics Committee for Research, Faculty of Medicine, Chiang Mai University, under the condition that both the subjects and their first degree relatives had given written informed consent prior to study participation.

### Subjects and criteria

We screened all DSM-IV schizophrenic patients, who visited the psychiatric clinic, for their eligibility to participate in the study. The inclusion criteria were patients aged 18 years old or more, who had taken antipsychotics for at least three months.

This study excluded schizophrenic patients who took medications for metabolic abnormalities. Recent findings have shown that new and emerging drug therapies for an individual component of MetS may affect others. While an antiobesity agent may reduce atherogenic dyslipidemia and abnormal glucose metabolism [12], many conventional antihypertensive drugs influence the development of new onset diabetes, both positively and negatively [13]. The inclusion of a patient receiving treatment for MetS or its component into a prospective study may therefore have an impact on the chance of developing other metabolic abnormalities.

Other exclusion criteria were i) hospitalization due to physical illnesses during 1 month prior to the visit, ii) pregnancy, and iii) regular renal hemodialysis.

### Assessment and outcomes

To assess the blood levels of triglycerides, HDL-cholesterol, and FPG, each subject had to fast himself/herself after midnight and have a blood test between 8 am and 10 am on the assessment day. Body weight was measured digitally with the subject wearing light cloth and no shoes. Waist circumference was measured in a horizontal plane, midpoint between the inferior margin of the ribs and the superior border of the iliac crest. Sitting blood pressure was measured digitally twice, and the mean reading was used.

All subjects received metabolic reassessment at 6 (range 5–7) months and 12 (range 11–13) months. Anyone who missed the 6-month assessment was still allowed to have a 12-month evaluation.

Only the IDF definition was applied to this study. This decision was made because the IDF is more purposefully developed for worldwide use than the updated ATP IIII. The IDF clearly defines the waist circumference cut points applied for South Asian/Chinese men and women living in Asia, who are closely related to Thais both ethnically and environmentally. The updated ATP III definition was not concurrently used because, as mentioned above, this diagnostic system is almost identical to the IDF one.

We clearly informed all subjects about the results of their metabolic assessment. For those who were found to have metabolic abnormalities, we gave them advice on a healthy lifestyle, including, moderate calorie restriction, moderate increase in physical activity and change in dietary composition. In addition, we encouraged them to report their metabolic results to their psychiatrists.

### Statistical analyses

The percentage of subjects who developed MetS was calculated. For the risk assessment, significant differences in proportions were determined by using Fisher's exact tests, and mean differences were assessed by using the Student-t tests. The p-values were two-tailed, and the term statistically significant applied to a p-value of < 0.05.

## Results

### Characteristics of the participants

Eighty-eight schizophrenic patients visited the clinic between February and August 2004. Twenty-five (28.4%) were excluded due to no consent given by the patients themselves and/or their 1^st ^degree relatives, and 6 (6.8%) because medications for metabolic disturbances had been taken. Fifty-seven patients (24 men and 33 women) participated in the study, with a mean ± SD age and duration of antipsychotic treatment of 37.5 ± 12.7 years old and 8.4 ± 6.3 years, respectively.

At baseline, 13 of the 57 participants (22.8%) fulfilled the MetS definition and were therefore excluded from the incidence analysis. Table 2 shows the demographic data and characteristics of all 57 subjects participating in the study, and the 44 subjects included in the incidence study.

**Table 2:** Demographic data and characteristics of the subjects in total, including those in the incidence study, and those who completed the incidence study^a, b^

	**Total participants (N = 57)**	**Subjects included the study (N = 44)**	**Subjects completed the study (N = 35)**
Number of male patients	24 (42.1)	18 (40.9)	13 (37.1)
Age, years	37.5 ± 12.7	35.5 ± 11.9	34.7 ± 11.2
Age at schizophrenia onset, years	28.5 ± 11.9	26.8 ± 11.1	26.0 ± 10.3
Duration of antipsychotic treatment	8.4 ± 6.3	8.2 ± 6.5	8.1 ± 6.3
Number of hospitalizations	1.8 ± 2.2	1.8 ± 2.5	1.6 ± 2.3
Patients taking second-generation antipsychotics^c ^(including, clozapine, olanzapine, risperidone, and sertindole)	33 (57.9)	24 (54.5)	19 (54.3)
Patients reporting metabolic disturbances in their 1^st ^degree relatives			
1. Obesity	6 (10.5)	5 (11.4)	3 (8.6)
2. Dyslipidemia	5 (8.8)	4 (9.1)	3 (8.6)
3. Hypertension	20 (35.1)	15 (34.1)	13 (37.1)
4. Diabetes mellitus	8 (14.0)	6 (13.6)	4 (11.4)
Patients with cluster diseases of MetS			
1. Obesity (BMI ≥ 30 kg/m^2^)	4 (7.0)	2 (4.5)	2 (5.7)
2. Central obesity (waist circumference ≥ 90 cm for men or ≥ 80 cm for women)	27 (47.4)	14 (31.8)	11 (31.4)
3. High triglyceride level (≥ 150 mg/dL)	22 (38.6)	12 (27.3)	10 (28.6)
4. Low HDL cholesterol level (<40 mg/dL for men or <50 mg/dL for women)	16 (28.1)	10 (22.7)	8 (22.9)
5. High blood pressure (≥ 130/≥ 85 mm Hg)	17 (29.8)	11 (25.0)	8 (22.9)
6. High fasting glucose level (≥ 100 mg/dL)	16 (28.1)	8 (18.2)	6 (17.1)

^a^Data shown as mean ± SD and N (%)

^b^No significant difference (p > 0.05) for all comparisons.

^c^The rest were taking first-generation antipsychotics.

Abbreviation: BMI = body mass index, HDL = high-density lipoprotein.

### Dropouts and the antipsychotics used

Of 44 subjects included in the incidence study, 9 (20.5%) were lost to follow-up. The demographic data and characteristics of the 35 subjects who completed the study were not significantly different from those of the 44 subjects included in the incidence study (see Table 2). At the end of a 1-year study, the number of subjects taking high-potency conventional antipsychotics was smaller, but that of participants receiving sertindole was larger. Table 3 shows the details of antipsychotic medications used at baseline and endpoint.

**Table 3:** Antipsychotics taken by subjects including those in the incidence study at baseline and endpoint

Medications	Number (%) of subjects taking at baseline (N = 44)	Number of subjects lost to follow-up (N = 9)	Number (%) of subjects taking at endpoint (N = 35)^a^
High-potency conventional antipsychotics^b^	12 (27.3)	2	4 (11.4)
Medium-potency conventional antipsychotics^c^	4 (9.1)	1	4 (11.4)
Low-potency conventional antipsychotics^d^	5 (11.4)	2	5 (14.3)
Clozapine	5 (11.4)	2	7 (20.0)
Olanzapine	3 (6.8)	1	5 (14.3)
Risperidone	14 (31.8)	1	13 (37.1)
Sertindole	1 (2.3)	0	12 (34.3)

^a^Including 7 patients developing metabolic syndrome and 28 patients not developing metabolic syndrome.

^b^High-potency conventional antipsychotics including haloperidol and trifluoperazine.

^c^Medium-potency conventional antipsychotics including clopenthixol and perphenazine.

^d^Low-potency conventional antipsychotics including chlorpromazine and thioridazine.

### Incidence analysis

Although the 44 subjects included in the analysis did not meet the definition at baseline, 16 of them had 1 component, 15 had 2 components, and 3 had 3 components. Only 10 of them (22.7%) had no metabolic abnormality defined by the IDF. Seven of the 35 subjects (20.0%) that could be followed up for one year developed IDF MetS. All 7 subjects who developed IDF MetS were those who already had 2 components at baseline.

### Risks of MetS

There was no significant difference between the 7 subjects who developed MetS and the 28 subjects who did not in respect of sex, age, age at schizophrenia onset, duration of antipsychotic treatment, number of hospitalizations, and self-reported family history of obesity, dyslipidemia, hypertension, and diabetes mellitus (see Table 4).

**Table 4:** Comparison the characteristics of patients developing and not developing MetS^a^

	Patients developing MetS (N = 7)	Patients not developing MetS (N = 28)	Significant difference
Number of male patients	3 (42.9)	9 (32.1)	Fisher's exact test, p = .68
Age, years	38.7 ± 12.9	35.3 ± 11.4	t = .69, df = 32, p = .49
Age at schizophrenia onset, years	27.0 ± 15.1	26.3 ± 8.8	t = .15, df = 32, p = .88
Duration of antipsychotic treatment	11.6 ± 8.7	8.1 ± 6.0	t = 1.25, df = 32, p = .22
Number of hospitalizations	2.1 ± 1.7	1.9 ± 3.0	t = .18, df = 32, p = -.86
Patients reporting metabolic disturbances in their 1^st ^degree relatives			
1. Obesity	1 (14.9)	2 (7.1)	Fisher's exact test, p = .51
2. Dyslipidemia	0 (0.0)	3 (10.7)	Fisher's exact test, p = 1.00
3. Hypertension	3 (42.9)	10 (35.7)	Fisher's exact test, p = .68
4. Diabetes mellitus	0 (0.0)	4 (14.3)	Fisher's exact test, p = .56

^a^Data shown as mean ± SD and N (%)

Abbreviation: MetS = metabolic syndrome.

## Discussion

This study found that 20.0% of Thai schizophrenic patients receiving long-term antipsychotic treatment developed MetS within a year of follow-up. However, no risk factor for its development could be found.

By applying the IDF MetS definition, metabolic abnormalities were very common in these Thai schizophrenic patients. After the exclusion of those receiving treatment for metabolic disturbances at baseline, 47 of 57 subjects (82.5%) included in this study still had metabolic abnormalities. Another interesting result was that all 7 subjects who developed MetS within the 12 months follow-up were part of the 15 participants who already had 2 MetS components at baseline. This finding may suggest that, for schizophrenic patients who have 2 MetS conditions, almost half of them may fulfill the MetS definition within a year.

To our knowledge, there has been no incidence study of MetS in schizophrenia patients. However, in comparison to the 3-year incidence rate of ATP III MetS in the general population in France (8.0%–10.5%) [14], our subjects may be more likely to develop MetS than the general population. However, due to ethnic and illness differences, this comparison should be viewed with great caution.

In this study, selection bias, a common problem found in epidemiological studies, was minimized by including all patients who visited our clinic. However, the switching of conventional antipsychotic medications to atypical ones in a large proportion of subjects may be the most important limitation of this study. Because some atypical antipsychotic agents have a worse metabolic profile than the conventional ones [15], the high rate of antipsychotic switching may directly influence the new-onset MetS in many studied subjects. In contrast, the interventions for metabolic abnormalities given during the study probably lower the actual rate of the incidence. Other limitations of this study include the exclusion of older patients with MetS at baseline, a large proportion of excluded patients, a small sample size, the lack of a control group, and a high drop-out rate.

The findings of this study may not be able to generalize in other ethnic groups. The differential prevalence of MetS across ethnic groups has been found in the general population [16] and schizophrenic patients [17]. It is therefore possible that the incidence rates of MetS also vary among ethnic groups with schizophrenia.

## Conclusion

Thai schizophrenic patients receiving long-term antipsychotic treatment are likely to have metabolic abnormalities. These abnormalities may rapidly progress and fulfill the MetS definition within years of follow-up. These findings support the importance of assessing and monitoring metabolic parameters in schizophrenic patients, as proposed recently [18,19].

## List of abbreviations used

ATP III = National Cholesterol Education Program Adult Treatment Panel III

FPG = fasting plasma glucose

HDL = high-density lipoprotein

IDF = International Diabetes Federation

MetS = metabolic syndrome

## Competing interests

The author(s) declare that they have no competing interests.

## Authors' contributions

MS conceived the study, participated in its design and coordination, performed the statistical analysis and drafted the manuscript. SL participated in the study design and coordination, and drafted the manuscript. VB participated in the study design and drafted the manuscript. CC participated in the study design and drafted the manuscript. NJ participated in the study design and drafted the manuscript. All authors read and approved the final manuscript.

## Pre-publication history

The pre-publication history for this paper can be accessed here:


